# Association between weight-adjusted-waist index and the prevalence of rheumatoid arthritis and osteoarthritis: a population-based study

**DOI:** 10.1186/s12891-023-06717-y

**Published:** 2023-07-20

**Authors:** Xiaohua Wang, Lin Xie, Shuo Yang

**Affiliations:** 1 Affiliated Hospital of Integrated Traditional Chinese and Western Medicine, Nanjing University of Chinese Medicine, Nanjing, China; 2 Jiangsu Province Academy of Traditional Chinese Medicine, Jiangsu, China

**Keywords:** Rheumatoid arthritis, NHANES, Obese, Osteoarthritis, Weight-adjusted-waist index, Adult

## Abstract

**Introduction:**

The weight-adjusted-waist Index (WWI), an innovative metric for assessing obesity, exhibits superior efficacy in appraising lean muscle and adipose tissue mass relative to both the Body Mass Index (BMI) and Waist Circumference (WC). The objective of this research paper is to investigate the correlation between WWI and the incidence of Rheumatoid Arthritis (RA) and Osteoarthritis (OA).

**Methods:**

In this population-based study, we collected data from adult participants aged 20–80 years using the National Health and Nutrition Examination Survey (NHANES) conducted between 2011 and 2020 to analyze the association between WWI and the occurrence of RA and OA. NHANES, a nationally representative cross-sectional survey, is designed to evaluate the health and nutritional status of the U.S. population. The current research incorporates an extensive, nationally representative sample of U.S. adults, utilizing weighted multivariate linear regression and smoothed curve fitting techniques to examine linear and non-linear relationships. Threshold effects were determined through a two-part linear regression model. Additionally, subgroup analyses and interaction tests were conducted to explore the connection between WWI and the incidence of RA and OA.

**Results:**

Our findings reveal a linear positive correlation between WWI and OA prevalence, indicating that an increase in WWI is linked to a heightened risk of OA. Conversely, a non-linear relationship was observed between WWI and RA prevalence, exhibiting a significant threshold effect with a saturation value of 11.21 cm/√kg. A positive association was detected to the left of the saturation point, while no significant association was present between the two variables to the right of the saturation point, suggesting a complex non-linear relationship between RA prevalence and WWI.

**Conclusions:**

This investigation demonstrates a positive linear association between WWI and OA prevalence, as well as a complex non-linear relationship with RA prevalence in U.S. adults aged 20–80 years.

## Background

Rheumatoid arthritis (RA) and osteoarthritis (OA) are two prevalent and debilitating musculoskeletal disorders with substantial impact on the quality of life ofaffected individuals [1, 2]. RA is an autoimmune, systemic inflammatory condition characterized by persistent inflammation of synovial joints, ultimately resulting in joint destruction and deformity [3]; it affects approximately 0.5–1% of the global population [4]. OA, on the other hand, is a degenerative joint disorder primarily involving synovial cartilage, subchondral bone, and synovial membrane [5]; it is the most common form of arthritis among over 100 types, afflicting around 3.8% of the world's population [6]. Despite being distinct types of arthritis, RA and OA exhibit some shared clinical features and a common inflammatory component, suggesting the presence of overlapping pathophysiological mechanisms [7, 8]. Research indicates that various genetic and environmental factors contribute to their development and progression [9]. Among these risk factors, some are difficult to modify, while others, such as obesity, are more susceptible to medical and behavioral interventions [10–12]. Obesity has long been recognized as a substantial risk factor for the onset and progression of OA, with a heightened body mass index (BMI) notably linked to the occurrence of knee and hip OA [13]. However, the association between obesity and RA is less clear, as some studies suggest a positive relationship, while others find no significant connection [14].

The Weight-Adjusted Waist Index (WWI) is an advanced anthropometric measure considered more accurate than waist circumference and BMI for appraising obesity-related health hazards [15, 16]. Investigating the correlation between WWI and the incidence of RA and OA may provide valuable understanding of the influence of central obesity on the pathogenesis of these conditions, ultimately contributing additional information for the prevention and management of OA and RA.

The National Health and Nutrition Examination Survey (NHANES) is a nationally representative cross-sectional investigation aimed at evaluating the health and nutritional status of the U.S. population [17]. To our knowledge, no research has yet established a link between WWI and the occurrence of OA and RA. As a result, this study seeks to analyze NHANES data from 2011 to 2020 to elucidate the association between WWI and the incidence of RA and OA.

## Methods

### Data source and participants

NHANES, a recurring cross-sectional investigation conducted biennially by the National Center for Health Statistics (NCHS) under the Centers for Disease Control and Prevention (CDC), utilizes a complex, multi-stage probability design to acquire a nationally representative sample of non-institutionalized U.S. civilians. The survey's objective is to assess the collective health status of the nation's population. NHANES participants submit written consent forms, which are reviewed and approved by the NCHS Ethics Review Committee, adhering to the principles outlined in the Declaration of Helsinki. The survey methodology comprises an in-depth household interview, followed immediately by a meticulous physical examination and blood collection at a dedicated Mobile Examination Center (MEC). Due to its comprehensive approach, NHANES data has been extensively employed for reliable estimations of the prevalence of diverse chronic diseases and their related risk factors. All NHANES data used in this study are publicly available at https://www.cdc.gov/nchs/nhanes/.

**Fig. 1:**
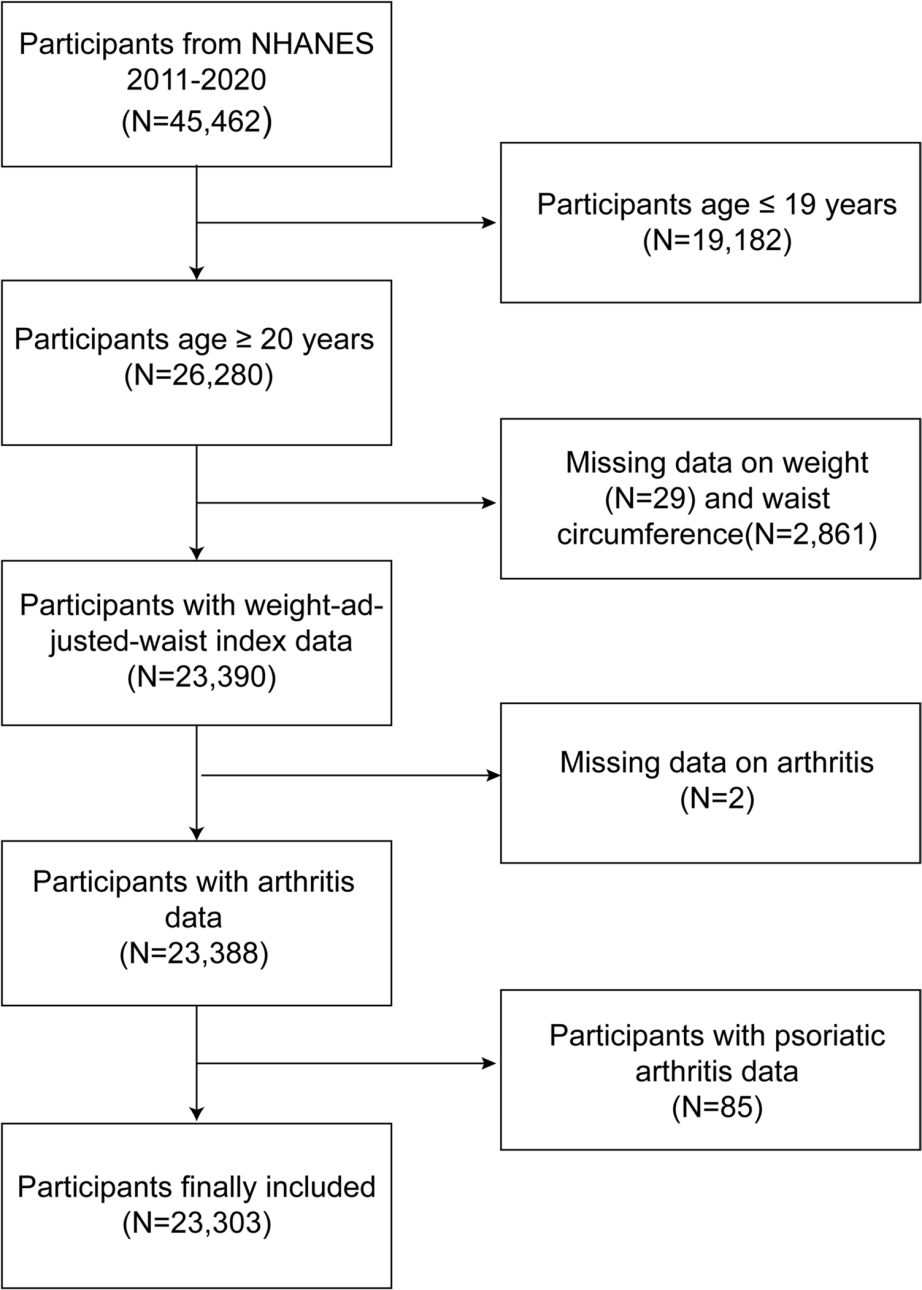
The nadir criteria for this study

In this analysis, a total of 45,462 participants were involved in five cycles (2011–2020) of the NHANES survey. Individuals below the age of 19 years (*n* = 19,182) were excluded. Furthermore, we omitted participants with missing data on waist circumference (*n* = 2,861), weight (*n* = 29), and arthritis information (*n* = 2). Additionally, we did not investigate the association between waist-to-weight index (WWI) and psoriatic arthritis, as the prevalence of psoriatic arthritis was relatively low (85 cases, 0.36%). Ultimately, this study incorporated a substantial, nationally representative sample (*n* = 23,303) of adults aged 20–80 years in the United States. A flow diagram illustrating the study is provided in Fig. 1 (The nadir criteria for this study).

## Collection and definition of data

In this study, the weight-adjusted waist index (WWI) was established as an exposure variable. WWI was computed by dividing each participant's waist circumference (WC) in centimeters by the square root of their weight in kilograms and rounding the result to two decimal places. To assess the prevalence of osteoarthritis and rheumatoid arthritis, we employed questionnaires featuring the following items:

- "Has a doctor ever told you that you have arthritis?"
- "What type of arthritis was it?"

The prevalence of osteoarthritis and rheumatoid arthritis was designated as the outcome variables.

The multivariate adjusted model encapsulates the potential association between the WWI index and osteoarthritis and rheumatoid arthritis. Covariates in our study encompassed:

- Age
- Sex
- Race
- Laboratory examinations [Alanine aminotransferase (ALT), Blood urea nitrogen (BUN), Total Calcium, Alkaline phosphatase,,Total Cholesterol, Aspartate aminotransferase (AST), Triglycerides, Low-density lipoprotein cholesterol (LDL-C), Uric acid, Direct high-density lipoprotein cholesterol (HDL-C)]
- Diabetes status
- Education level
- Smoking status
- Alcohol consumption
- Sleep disorders
- Moderate recreational activities
- Working hours

For supplementary information regarding confounding factors, please visit: http://www.cdc.gov/nchs/nhanes/.

## Statistical analysis

Statistical analyses in this study were performed using R (http://www.r-project.org) and EmpowerStats (http://www.empowerstats.com), with a significance threshold of P < 0.05. Considering NHANES aims to generate data representative of the non-institutionalized civilian population in the United States, all estimates were calculated using sample weights following NCHS analysis guidelines. Weighted multiple linear regression analysis was applied to explore the linear correlation between WWI and the incidence of RA and OA. In contrast, smoothed curve fitting and threshold effect evaluation were employed to assess the non-linear association between WWI and the occurrence of RA and OA. The study encompassed three models: Model 1 remained unadjusted for variables, Model 2 was adjusted for sex, race and age, and Model 3 was adjusted for all covariates. Subgroup analyses were additionally carried out.

## Results

### Baseline characteristics

Upon applying the inclusion and exclusion criteria, 23,303 participants were incorporated into the study, with a mean age of 49.29 ± 17.43 years. The sample consisted of 48.8% males and 51.2% females, as well as 36.52% non-Hispanic white individuals, 23.85% non-Hispanic black individuals, 12.95% Mexican–American individuals, 10.68% other Hispanic individuals, and 16.00% individuals from other racial backgrounds. The mean (SD) value of the WWI was 11.09 (0.86) cm/√kg. The number and proportion of participants with osteoarthritis and rheumatoid arthritis were 2,670 (11.46%) and 1,201 (5.15%), respectively. Table 1 summarizes the clinical characteristics of participants, with stratified groups based on WWI quartiles (Q) displayed in the columns. Participants in the highest quartile group of the WWI tended to be older, female, non-Hispanic white, non-alcoholic, non-smokers, engaged in more recreational activities, did not have sleep disorders, worked fewer than 35 h per week, possessed a high school education or higher, and did not have diabetes, arthritis, rheumatoid arthritis, or osteoarthritis. Our analysis revealed significant differences between the studied groups. Specifically, they manifested elevated levels of alanine aminotransferase (ALT), aspartate aminotransferase (AST), alkaline phosphatase (ALP), uric acid, total cholesterol, triglycerides, low-density lipoprotein cholesterol (LDL-C), Body Mass Index (BMI), weight, and waist circumference (WC). Conversely, these groups displayed lower measures of height, Poverty Income Ratio (PIR), total calcium, and direct high-density lipoprotein cholesterol (HDL-C). It is thus clear from our data that there are considerable disparities among the groups under examination. (Table 1).

**Table 1:** Basic characteristics of participants by weight-adjusted-waist index quartile

**Characteristics**	**Weight-adjusted-waist index(cm/√kg)**			
	**Q1 (8.37–9.97)** ***N* = 5826**	**Q2 (9.97–10.62)** ***N* = 5825**	**Q3 (10.62–11.26)** ***N* = 5825**	**Q4 (11.26–14.79)** ***N* = 5827**
**Age**	37.69 ± 14.16	47.17 ± 15.60	53.28 ± 16.27	59.04 ± 16.04
**Sex**				
Male	3551 (60.95%)	3150 (54.08%)	2768 (47.52%)	1903 (32.66%)
Female	2275 (39.05%)	2675 (45.92%)	3057 (52.48%)	3924 (67.34%)
**Races**				
Mexican American	420 (7.21%)	708 (12.15%)	931 (15.98%)	959 (16.46%)
Other Hispanic	447 (7.67%)	616 (10.58%)	711 (12.21%)	715 (12.27%)
Non-Hispanic White	2030 (34.84%)	2010 (34.51%)	2072 (35.57%)	2398 (41.15%)
Non-Hispanic Black	1824 (31.31%)	1378 (23.66%)	1265 (21.72%)	1091 (18.72%)
Other Race	1105 (18.97%)	1113 (19.11%)	846 (14.52%)	664 (11.40%)
**Laboratory examination**				
ALT(U/L)	22.43 ± 17.45	25.08 ± 19.41	25.40 ± 18.60	23.91 ± 23.76
AST(U/L)	24.13 ± 14.85	24.39 ± 13.54	24.70 ± 17.87	24.32 ± 18.32
ALP (IU/L)	63.93 ± 20.36	68.97 ± 21.81	73.58 ± 23.71	78.23 ± 30.30
BUN(mg/dL)	12.97 ± 4.34	13.68 ± 5.11	14.20 ± 5.81	15.34 ± 7.07
Total Calcium (mg/dL)	9.39 ± 0.33	9.34 ± 0.35	9.34 ± 0.37	9.33 ± 0.38
Uric acid (mg/dL)	5.18 ± 1.30	5.38 ± 1.38	5.47 ± 1.43	5.60 ± 1.44
Total Cholesterol (mg/dL)	182.20 ± 36.46	192.33 ± 39.92	192.46 ± 41.67	190.17 ± 42.53
Triglyceride (mg/dL)	105.20 ± 59.51	116.80 ± 72.13	121.91 ± 80.66	124.36 ± 64.11
LDL-C (mg/dL)	108.84 ± 22.35	112.61 ± 23.84	112.24 ± 24.78	110.85 ± 24.47
Direct HDL-C(mg/dL)	57.04 ± 16.00	53.41 ± 16.18	51.81 ± 15.66	50.86 ± 14.09
**Diabetes status**				
Yes	164 (2.81%)	520 (8.93%)	900 (15.45%)	1578 (27.08%)
No	5588 (95.91%)	5179 (88.91%)	4709 (80.84%)	4040 (69.33%)
Borderline	74 (1.27%)	126 (2.16%)	216 (3.71%)	209 (3.59%)
**Education level**				
Less than high school	754 (12.94%)	1073 (18.42%)	1397 (23.98%)	1681 (28.85%)
High school	1185 (20.34%)	1274 (21.87%)	1377 (23.64%)	1421 (24.39%)
Above high school	3887 (66.72%)	3478 (59.71%)	3051 (52.38%)	2725 (46.77%)
**Smoking status**				
Now	2129 (36.54%)	2230 (38.28%)	2055 (35.28%)	1998 (34.29%)
Former	436 (7.48%)	518 (8.89%)	565 (9.70%)	713 (12.24%)
Never	3261 (55.97%)	3077 (52.82%)	3205 (55.02%)	3116 (53.48%)
**Alcohol abuse (Ever have 4/5 or more drinks every day)**				
Yes	831 (14.26%)	964 (16.55%)	1042 (17.89%)	992 (17.02%)
No	4995 (85.74%)	4861 (83.45%)	4783 (82.11%)	4835 (82.98%)
**Sleep disorders**				
Yes	1091 (18.73%)	1424 (24.45%)	1675 (28.76%)	2037 (34.96%)
No	4735 (81.27%)	4401 (75.55%)	4150 (71.24%)	3790 (65.04%)
**Moderate recreational activities**				
Yes	2979 (51.13%)	2589 (44.45%)	2325 (39.91%)	1862 (31.95%)
No	2847 (48.87%)	3236 (55.55%)	3500 (60.09%)	3965 (68.05%)
**Arthritis**				
No	5200 (89.26%)	4605 (79.06%)	4029 (69.17%)	3324 (57.04%)
Yes	626 (10.74%)	1220 (20.94%)	1796 (30.83%)	2503 (42.96%)
**Osteoarthritis**				
No	5559 (95.42%)	5307 (91.11%)	5070 (87.04%)	4697 (80.61%)
Yes	267 (4.58%)	518 (8.89%)	755 (12.96%)	1130 (19.39%)
**Rheumatoid arthritis**				
No	5704 (97.91%)	5586 (95.90%)	5448 (93.53%)	5364 (92.05%)
Yes	122 (2.09%)	239 (4.10%)	377 (6.47%)	463 (7.95%)
**Working hours (Usually work 35 or more hours per week)**				
Yes	2190 (37.59%)	2261 (38.82%)	2121 (36.41%)	1994 (34.22%)
No	3636 (62.41%)	3564 (61.18%)	3704 (63.59%)	3833 (65.78%)
**PIR**	2.67 ± 1.61	2.69 ± 1.57	2.49 ± 1.54	2.22 ± 1.43
**Physical examination**				
Weight (kg)	74.07 ± 17.08	80.72 ± 19.90	84.36 ± 22.04	88.91 ± 24.89
BMI (kg/m^2^)	24.97 ± 4.81	28.26 ± 5.62	30.47 ± 6.52	33.74 ± 7.82
Height (m)	1.72 ± 0.09	1.68 ± 0.09	1.66 ± 0.09	1.62 ± 0.09
Waist Circumference (cm)	85.50 ± 10.71	96.41 ± 11.89	103.59 ± 13.41	113.75 ± 16.15
WWI (cm/√kg)	9.99 ± 0.40	10.81 ± 0.17	11.37 ± 0.17	12.18 ± 0.42

Mean ± SD for continuous variables

*Abbreviation*: *Q* quartile, *PIR* ratio of family income to poverty, *BMI* body mass index, *LDL-C* low-density lipoprotein cholesterol, *BMD* bone mineral density, *HDL-C* high-density lipoprotein cholesterol, *AST* aspartate aminotransferase, *ALT* alanine aminotransferase, *ALP* alkaline phosphatase, *BUN* blood urea nitrogen

### Association between WWI and osteoarthritis prevalence

Table 2 illustrates the relationship between WWI and osteoarthritis. In comparison to the reference level (Q1), the association between WWI and osteoarthritis was significantly positive in Model 1 without adjustment for covariates [OR = 2.03 (1.74, 2.37) for Q2, OR = 3.10 (2.68, 3.58) for Q3, and OR = 5.01 (4.36, 5.76) for Q4]. Furthermore, after adjusting for covariates such as gender, age, and race, this positive association remained (P > 0.05), with an OR of 1.57 (1.34, 1.84) for Quartile 4. Additionally, after adjusting for other covariates, this positive association persisted, with an OR of 1.37 (1.16, 1.63) for Quartile 4.

**Table 2:** Association between weight-adjusted-waist index (cm/√kg) and osteoarthritis prevalence

Exposure	Model 1 [OR (95% CI)]	Model 2 [OR (95% CI)]	Model 3 [OR (95% CI)]
WWI (quartile)			
Quartile 1	reference	reference	reference
Quartile 2	2.03 (1.74, 2.37)	1.26 (1.07, 1.48)	1.17 (0.99, 1.38)
Quartile 3	3.10 (2.68, 3.58)	1.39 (1.19, 1.63)	1.26 (1.07, 1.48)
Quartile 4	5.01 (4.36, 5.76)	1.57 (1.34, 1.84)	1.37 (1.16, 1.63)
P for trend	< 0.0001	< 0.0001	0.0002

Model 1: no covariates were adjusted. Model 2: age;gender;race were adjusted. Model 3: age; gender; race; diabetes; PIR; ALT; AST; ALP; BUN; total calcium; uric acid; total cholesterol; triglyceride; LDL-C; direct HDL-C; diabetes status; education level; smoking status; alcohol abuse; sleep disorders; moderate recreational activities were adjusted

*Abbreviation*: *PIR* ratio of family income to poverty, *BMI* body mass index, *LDL-C* low-density lipoproteincholesterol, *HDL-C* high-density lipoprotein cholesterol, *AST* aspartate aminotransferase, *ALT* alanine aminotransferase, *ALP* alkaline phosphatase, *BUN* blood urea nitrogen

**Fig. 2:**
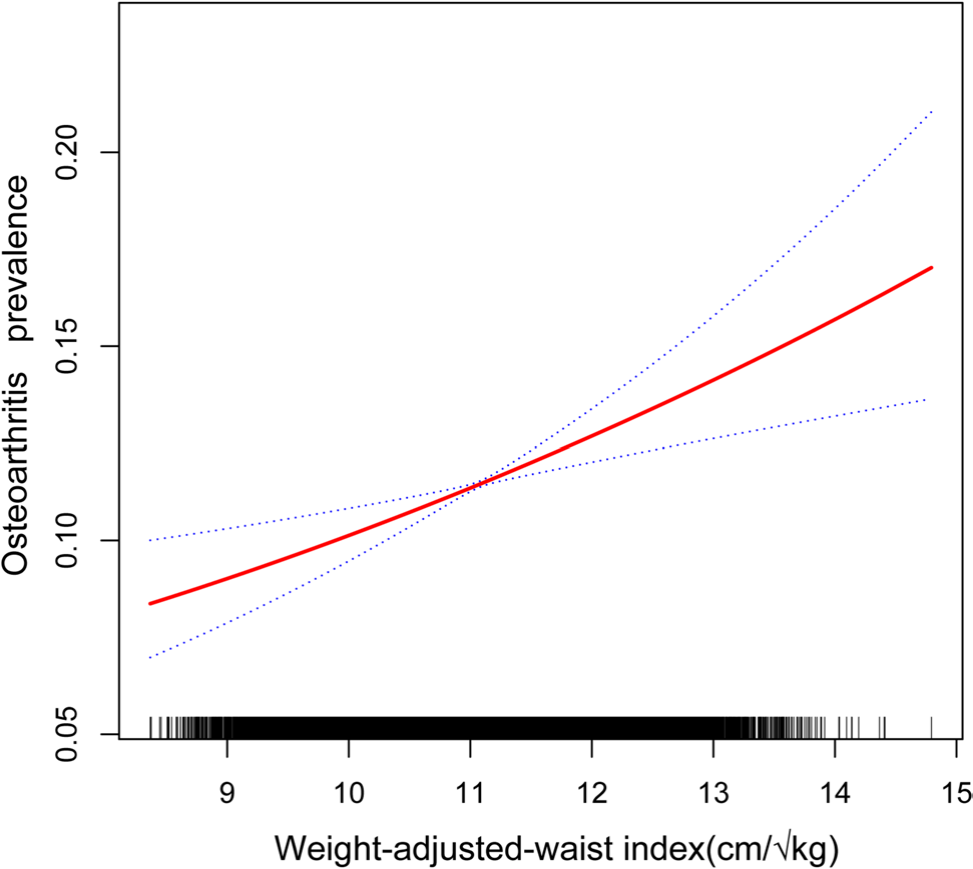
Association between WWI and osteoarthritis prevalence

Moreover, the smooth curve fitting results revealed no discernible non-linear association between WWI and OA prevalence (Fig. 2: Association between WWI and osteoarthritis prevalence).

### Association between WWI and rheumatoid arthritis prevalence

Similarly, Table 3 displays the relationship between WWI and rheumatoid arthritis. In comparison to the reference level (Q1), the association between WWI and rheumatoid arthritis was significantly positive in Model 1 without adjustment for covariates [OR = 2.00 (1.60, 2.50) for Q2, OR = 3.24 (2.63, 3.98) for Q3, and OR = 4.04 (3.29, 4.94) for Q4]. Furthermore, after adjusting for covariates such as gender, age, and race, this positive association remained (P > 0.05), with an OR of 2.02 (1.62, 2.54) for Quartile 4. Additionally, after adjusting for other covariates, this positive association persisted, with an OR of 1.43 (1.13, 1.82) for Quartile 4.

**Table 3:** Association between weight-adjusted-waist index (cm/√kg) and rheumatoid arthritis prevalence

Exposure	Model 1 [OR (95% CI)]	Model 2 [OR (95% CI)]	Model 3 [OR (95% CI)]
WWI (quartile)			
Quartile 1	reference	reference	reference
Quartile 2	2.00 (1.60, 2.50)	1.49 (1.19, 1.87)	1.34 (1.07, 1.69)
Quartile 3	3.24 (2.63, 3.98)	1.95 (1.57, 2.43)	1.60 (1.27, 2.00)
Quartile 4	4.04 (3.29, 4.94)	2.02 (1.62, 2.54)	1.43 (1.13, 1.82)
P for trend	< 0.0001	< 0.0001	0.0145

Model 1: no covariates were adjusted. Model 2: age;gender;race were adjusted. Model 3: age; gender; race; diabetes; PIR; ALT; AST; ALP; BUN; total calcium; uric acid; total cholesterol; triglyceride; LDL-C; direct HDL-C; diabetes status; education level; smoking status; alcohol abuse; sleep disorders; moderate recreational activities were adjusted

*Abbreviation*: *PIR* ratio of family income to poverty, *BMI* body mass index, *LDL-C* low-density lipoproteincholesterol, *HDL-C* high-density lipoprotein cholesterol, *AST* aspartate aminotransferase, *ALT* alanine aminotransferase, *ALP* alkaline phosphatase, *BUN* blood urea nitrogen

**Fig. 3:**
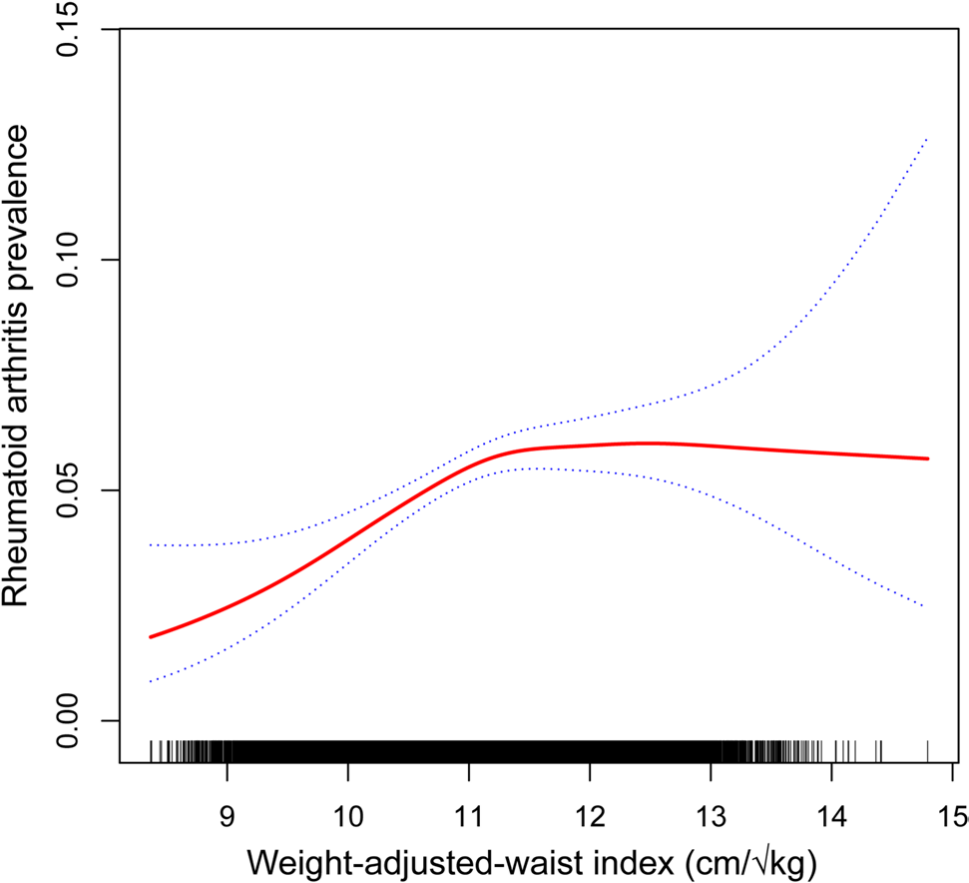
Association between WWI and rheumatoid arthritis prevalence

Conversely, the smooth curve fitting results indicated a non-linear association between WWI and RA prevalence (Fig. 3: Association between WWI and rheumatoid arthritis prevalence).

### Subgroup analysis between WWI and osteoarthritis prevalence

No significant differences were observed between WWI and osteoarthritis prevalence in other subgroup strata, except for race and working hours (P for interaction > 0.05, Table 4). Specifically, the results indicate that the positive association between WWI and osteoarthritis prevalence was consistently robust in subgroup analyses stratified by age, sex, education, alcohol abuse, smoking, appropriate recreational activity, and sleep disturbance (Table 4). Moreover, non-Hispanic black participants and those who worked more than 35 h per week exhibited a higher risk of osteoarthritis with increased WWI compared to other races and those who worked fewer than 35 h per week (p < 0.001); however, this effect size was not significant within each group.

**Table 4:** Association between weight-adjusted-waist index (cm/√kg) and osteoarthritis prevalence

	***OR* (95*%* CI)**	***P* for interaction**
**Stratified by gender**		0.3371
Males	1.20 (1.07, 1.35)	
Females	1.12 (1.03, 1.21)	
**Stratified by race**		0.0400
Mexican American	1.06 (0.82, 1.35)	
Other Hispanic	1.05 (0.82, 1.33)	
Non-Hispanic White	1.05 (0.96, 1.16)	
Non-Hispanic Black	1.37 (1.19, 1.57)	
Other Race	1.16 (0.93, 1.45)	
**Stratified by age**		0.2178
< 65	0.34 (0.20, 0.48)	
≥ 65	0.24 (0.17, 0.31)	
**Stratified by diabetes**		0.3539
Yes	1.18 (1.02, 1.37)	
No	1.13 (1.05, 1.22)	
Borderline	0.91 (0.66, 1.26)	
**Stratified by education level**		0.8132
Less than high school	1.18 (1.02, 1.37)	
High school	1.13 (0.99, 1.30)	
Above high school	1.12 (1.03, 1.22)	
**Stratified by smoking status**		0.3015
Now	1.26 (1.02, 1.55)	
Former	1.15 (1.05, 1.26)	
Never	1.06 (0.95, 1.19)	
**Stratified by alcohol abuse**		0.0588
Yes	1.17 (1.08, 1.25)	
No	0.98 (0.83, 1.16)	
**Stratified by sleep disorders**		0.3993
Yes	1.16 (1.06, 1.26)	
No	1.10 (1.00, 1.21)	
**Stratified by working hours (Usually work 35 or more hours per week)**		0.0268
Yes	1.19 (1.10, 1.28)	
No	1.01 (0.90, 1.14)	
**Stratified by moderate recreational activities**		0.2370
Yes	1.08 (0.97, 1.20)	
No	1.17 (1.08, 1.27)	

In subgroup analyses stratified by sex, race, age, diabetes status,education level; smoking status, alcohol abuse, sleep disorders, moderate recreational activities, The model adjusted for covariates such age; gender; race; diabetes; PIR; ALT; AST; ALP; BUN; total calcium; uric acid; total cholesterol; triglyceride; LDL-C; direct HDL-C; diabetes status; education level; smoking status; alcohol abuse; sleep disorders; moderate recreationalactivities, but the modeldid not adjust for the stratification variables themselves

*Abbreviation*: *PIR* ratio of family income to poverty, *BMI* body mass index, *LDL-C* low-density lipoproteincholesterol, *HDL-C* high-density lipoprotein cholesterol, *AST* aspartate aminotransferase, *ALT* alanine aminotransferase, *ALP* alkaline phosphatase, *BUN* blood urea nitrogen

### Subgroup analysis between WWI and rheumatoid arthritis prevalence

No significant differences were observed between WWI and rheumatoid arthritis prevalence in other subgroup strata, except for age (P for interaction > 0.05, Table 5). Specifically, the results demonstrated that the positive association between WWI and rheumatoid arthritis prevalence was consistently robust in subgroup analyses stratified by age, sex, race, education, alcohol use, smoking, appropriate recreational activities, sleep disturbance, and work hours (Table 5). Additionally, participants aged < 65 years exhibited a higher risk of rheumatoid arthritis than those aged > 65 years with increased WWI (*p* < 0.001).

**Table 5:** Association between weight-adjusted-waist index (cm/√kg) and rheumatoid arthritis prevalence

	***OR* (95*%* CI)**	***P* for interaction**
**Stratified by gender**		0.2617
Males	1.21 (1.05, 1.41)	
Females	1.10 (0.98, 1.23)	
**Stratified by race**		0.3966
Mexican American	1.41 (1.08, 1.85)	
Other Hispanic	1.06 (0.80, 1.41)	
Non-Hispanic White	1.22 (1.05, 1.42)	
Non-Hispanic Black	1.07 (0.92, 1.25)	
Other Race	1.07 (0.77, 1.48)	
**Stratified by age**		0.0024
<65	0.43 (0.27, 0.59)	
≥ 65	0.14 (0.04, 0.24)	
**Stratified by diabetes**		0.8234
Yes	1.19 (0.99, 1.44)	
No	1.14 (1.02, 1.27)	
Borderline	1.03 (0.64, 1.65)	
**Stratified by education level**		0.6737
Less than high school	1.10 (0.93, 1.30)	
High school	1.22 (1.03, 1.45)	
Above high school	1.13 (0.99, 1.28)	
**Stratified by smoking status**		0.3642
Now	1.21 (1.07, 1.38)	
Former	1.07 (0.93, 1.24)	
Never	1.05 (0.80, 1.39)	
**Stratified by alcohol abuse**		0.1062
Yes	1.18 (1.07, 1.31)	
No	0.98 (0.80, 1.20)	
**Stratified by sleep disorders**		0.1973
Yes	1.20 (1.06, 1.35)	
No	1.06 (0.93, 1.22)	
**Stratified by working hours (Usually work 35 or more hours per week)**		0.3751
Yes	1.20 (1.04, 1.39)	
No	1.12 (1.01, 1.24)	
**Stratified by moderate recreational activities**		0.4829
Yes	1.10 (0.96, 1.27)	
No	1.17 (1.05, 1.30)	

In subgroup analyses stratified by sex, race, age, diabetes status,education level; smoking status, alcohol abuse, sleep disorders, moderate recreational activities, The model adjusted for covariates such age; gender; race; diabetes; PIR; ALT; AST; ALP; BUN; total calcium; uric acid; total cholesterol; triglyceride; LDL-C; direct HDL-C; diabetes status; education level; smoking status; alcohol abuse; sleep disorders; moderate recreationalactivities, but the modeldid not adjust for the stratification variables themselves

*Abbreviation*: *PIR* ratio of family income to poverty, *BMI* body mass index, *LDL-C* low-density lipoproteincholesterol, *HDL-C* high-density lipoprotein cholesterol, *AST* aspartate aminotransferase, *ALT* alanine aminotransferase, *ALP* alkaline phosphatase, *BUN* blood urea nitrogen

### Non-linearity and saturation effect analysis between WWI and rheumatoid arthritis prevalence

The smooth curve fit disclosed a non-linear association between WWI and rheumatoid arthritis (Fig. 3). Additionally, a breakpoint (K) of 11.21 (cm/√kg) was determined through saturation effect analysis. To the left of the breakpoint, a positive correlation between WWI and rheumatoid arthritis prevalence was observed (OR = 1.53, 95% CI 1.26–1.86; *P* for trend < 0.0001). Conversely, no statistically significant association between WWI and rheumatoid arthritis was identified to the right of the breakpoint (OR = 0.97, 95% CI 0.85, 1.11; P for trend = 0.6473). The log-likelihood ratio test *P* value was < 0.001 (Table 6).

**Table 6:** Saturation effect analysis of WWI(cm/√kg) on rheumatoid arthritis prevalence

	Model:Saturation effect analysis
WWI turning point (K)	11.21
< K,effect1	1.53 (1.26, 1.86) < 0.0001
> K,effect2	0.97 (0.85, 1.11) 0.6473
Log-likelihood ratio	< 0.001

Age; gender; race; diabetes; PIR; ALT; AST; ALP; BUN; total calcium; uric acid; total cholesterol; triglyceride; LDL-C; direct HDL-C; diabetes status; education level; smoking status; alcohol abuse; sleep disorders; moderate recreational activities were adjusted

*Abbreviation*: *PIR* ratio of family income to poverty, *BMI* body mass index, *LDL-C* low-density lipoproteincholesterol, *HDL-C* high-density lipoprotein cholesterol, *AST* aspartate aminotransferase, *ALT* alanine aminotransferase, *ALP* alkaline phosphatase, *BUN* blood urea nitrogen

## Discussion

The primary findings of our study can be summarized as follows: First, a significant positive linear correlation was identified between WWI and OA prevalence, indicating that an increase in WWI is strongly associated with a rise in OA prevalence. Second, a non-linear relationship was discovered between WWI and RA prevalence, with distinct associations between RA prevalence and WWI observed on the left and right sides of the breakpoint (WWI = 11.21 cm/√kg). WWI was positively associated with RA prevalence on the left side of the breakpoint, while the association on the right side of the breakpoint was not statistically significant.

The Weight-adjusted-waist index (WWI) offers numerous advantages over the traditional Body Mass Index (BMI) in assessing individual health risk factors. By accounting for waist circumference—a direct measure of visceral fat—the WWI addresses the shortcoming of BMI that might overlook individuals with excess body fat who have a normal overall weight. WWI effectively captures critical health-related changes such as age-related shifts from muscle mass to fat, particularly around the waist, which can often occur without significant changes in overall weight, leading to an unchanged BMI. Moreover, the WWI better accommodates gender-specific weight distributions and varying fat distribution patterns across ethnicities, facilitating a more nuanced risk assessment. Notwithstanding the practical challenges associated with waist measurements required by WWI, its proficiency in capturing comprehensive health profiles reinforces its superiority. However, it should not be used in isolation, but as a part of a wider health assessment strategy, and should always be complemented by professional medical advice. While discussing the WWI, it's vital to note a crucial conceptual distinction—the term, in a stricter sense, should be 'Body Mass-adjusted-waist index' as we are assessing body mass rather than weight, with 'weight' being a vector quantity representing both magnitude and direction. However, in medical and public health literature, 'weight' is conventionally used to denote body mass measured on a scale, leading to the prevalence of the term WWI. For consistency with existing literature and common usage, we use WWI, but we acknowledge this conceptual distinction and advocate for mindful usage and interpretation of such terms in health-related research and practice.

To the best of our knowledge, this is the premier cross-sectional study examining the association between WWI and the prevalence of OA and RA. Widely accepted criteria for defining obesity encompass BMI and WC [17, 18]. Numerous investigations have identified a positive correlation between BMI, WC, and the incidence of OA and RA. On one hand, a meta-analysis by Jiang et al., incorporating 21 studies, deduced that BMI was significantly and positively correlated with OA prevalence, with a 5-unit increase in BMI associated with a 35% rise in OA prevalence [19]. However, this meta-analysis also exhibited considerable heterogeneity, warranting further studies for validation. Moreover, Park et al. discovered that elevated BMI and larger WC augmented the risk of knee OA in a dose-dependent manner, analyzing data from 1,139,463 adults aged 50 years and older sourced from the Korean National Health Insurance Service (KNHIS) and classifying them as generally obese and centrally obese based on BMI and WC, respectively [20]. On the other, Marchand et al. incorporated 108,505 participants aged 25–42 years without RA from The Nurses' Health Study II (NHSII) and conducted a 25-year follow-up survey from 1989 to 2015. They ultimately inferred that long-term weight gain was strongly associated with an increased risk of RA, with weight gain ≥ 20 kg linked to over a threefold increased RA risk [21]. Additionally, Fergusond et al. utilized UK Biobank data to include 502,682 participants aged 40–70 years in a cross-sectional study, establishing that a higher waist circumference correlated with a higher rheumatoid arthritis prevalence. Importantly, this relationship persisted after adjusting for BMI, highlighting the potential significance of central obesity in autoimmune diseases such as RA. [22]. However, as research has progressed, some scholars have identified an obesity paradox when using BMI and WC as obesity measures [23–25]. The obesity paradox suggests that obesity does not necessarily shorten patients' expected survival time, and that overweight individuals may have a slightly lower risk of death compared to those of normal weight, and in some cases, may even exhibit beneficial effects. This paradox has led researchers to question the validity of BMI and WC as obesity measures. Consequently, it is crucial to identify an obesity index that eliminates the obesity paradox. The WWI is a recently developed anthropometric index considered a reliable obesity measure, in addition to BMI and WC, due to its simplicity of calculation and ability to differentiate between lean and fat [26, 27]. Contemporary studies have demonstrated the WWI's capacity to distinguish between lean and fat, and its application has expanded to various areas, including bone metabolism-related disorders and obesity. In our study, we discovered a linear positive correlation between WWI and OA prevalence, and a non-linear relationship with RA prevalence, with a breakpoint of 11.21, indicating a significant threshold effect between WWI and RA prevalence.

Obesity has been associated with the development and progression of osteoarthritis (OA) and rheumatoid arthritis (RA); however, the underlying mechanisms are multifaceted and complex [28]. In OA, the mechanical loading hypothesis posits that being overweight places an increased burden on weight-bearing joints, particularly the knee and hip, leading to cartilage degeneration and subsequent joint damage [29]. Moreover, adipose tissue is believed to function as an active endocrine organ, secreting adipokines such as leptin and adiponectin, which contribute to synovial inflammation, cartilage breakdown, and matrix metalloproteinase production, thereby exacerbating OA's pathological changes [30]. In the context of RA, obesity may contribute to its pathogenesis by fostering a chronic pro-inflammatory state, characterized by elevated levels of tumor necrosis factor-α (TNF-α) and interleukin-6 (IL-6), which in turn stimulate autoantibody production and inflammatory cell activation [31]. Moreover, obesity is related to an increased risk of developing RA, potentially due to adipokine dysregulation and impaired resolution of inflammation, resulting in persistent synovitis and joint destruction [32]. Besides the molecular and cellular mechanisms mentioned earlier, obesity has also been connected to the activation of the nucleotide-binding oligomerization domain-like receptor protein 3 (NLRP3) inflammasome, a critical intracellular signaling complex involved in the innate immune response [33, 34]. The NLRP3 inflammasome promotes the production of pro-inflammatory cytokines, such as IL-1β and IL-18, which further exacerbate OA and RA through synovial inflammation and cartilage degeneration [35]. Furthermore, activation of the NLRP3 inflammasome has been shown to be associated with obesity-induced insulin resistance, which may indirectly influence the pathogenesis of OA and RA by aggravating systemic inflammation [36, 37]. Additionally, the role of the gut microbiome in the development of OA and RA should not be underestimated. Obesity is linked to dysbiosis of the gut microbiota, leading to increased intestinal permeability and the translocation of bacterial lipopolysaccharides (LPS) into circulation, thus provoking systemic inflammation. Emerging evidence suggests that the gut microbiota may contribute to the pathogenesis of OA and RA by modulating host immune responses, inflammation, and metabolism [38–40].

Our study has several strengths and limitations. The primary strengths include the following: first, the sample size in this study was sufficiently large, providing greater statistical power and enhancing the reliability of the findings. Second, the study adjusted for numerous potential confounders to ensure more accurate associations between risk factors and the prevalence of OA and RA. Third, we identified a non-linear relationship between WWI and RA risk, offering additional evidence for a threshold effect. This study also presents several limitations. First, the cross-sectional nature of the study hinders the establishment of causality and may be influenced by confounding factors. Second, relying on self-reported diagnoses of OA and RA might reduce the accuracy of prevalence estimates, although previous studies have confirmed the acceptable accuracy of questionnaires. The study focused on the US population and did not consider variations in prevalence and risk factors across countries and ethnicities. As only participants from one ethnically limited country were included, generalizing our findings may be inappropriate.

## Conclusion

The findings of this investigation indicate a linear positive association between WWI and OA prevalence, as well as a non-linear relationship with RA prevalence among US adults, demonstrating a significant threshold effect. Nonetheless, further longitudinal studies incorporating a more diverse cohort are necessary to validate these results.

## Data Availability

The survey data are publicly available on the internet for data users and researchers throughout the world (www.cdc.gov/nchs/nhanes/).

## Abbreviations

WWI: Weight-Adjusted Waist Index
BMI: Body mass index
WC: Waist circumference
BMD: Bone mineral density
OP: Osteoporosis
PBM: Peak bone mass
NHANES: National Health and Nutrition Examination Survey
ALP: Alkaline phosphatase
AST: Aspartate aminotransferase
HDL-C: High-density lipoprotein cholesterol
LDL-C: Low-density lipoprotein cholesterol
PIR: Ratio of family income to poverty
BUN: Blood urea nitrogen
NCHS: National Center for Health Statistics
